# Hierarchical contribution of Argonaute proteins to antiviral protection

**DOI:** 10.1093/jxb/erad327

**Published:** 2023-08-21

**Authors:** Márta Ludman, Gabriella Szalai, Tibor Janda, Károly Fátyol

**Affiliations:** Institute of Genetics and Biotechnology, Hungarian University of Agriculture and Life Sciences, Szent-Györgyi Albert u. 4. Gödöllő 2100Hungary; Department of Plant Physiology and Metabolomics, Agricultural Institute, Centre for Agricultural Research, ELKH, Brunszvik u. 2. Martonvásár 2462Hungary; Department of Plant Physiology and Metabolomics, Agricultural Institute, Centre for Agricultural Research, ELKH, Brunszvik u. 2. Martonvásár 2462Hungary; Institute of Genetics and Biotechnology, Hungarian University of Agriculture and Life Sciences, Szent-Györgyi Albert u. 4. Gödöllő 2100Hungary; Nanjing Agricultural University, China

**Keywords:** Antiviral RNAi, Argonaute proteins, CRISPR/Cas9, genome editing, *Nicotiana benthamiana*, pepino mosaic virus, plant virus, RDR6, vsiRNA

## Abstract

Antiviral RNAi is the main protective measure employed by plants in the fight against viruses. The main steps of this process have been clarified in recent years, primarily relying on the extensive genetic resources of *Arabidopsis thaliana*. Our knowledge of viral diseases of crops, however, is still limited, mainly due to the fact that *A. thaliana* is a non-host for many agriculturally important viruses. In contrast, *Nicotiana benthamiana* has an unparalleled susceptibility to viruses and, since it belongs to the *Solanaceae* family, it is considered an adequate system for modeling infectious diseases of crops such as tomatoes. We used a series of *N. benthamiana* mutants created by genome editing to analyze the RNAi response elicited by the emerging tomato pathogen, pepino mosaic virus (PepMV). We uncovered hierarchical roles of several Argonaute proteins (AGOs) in anti-PepMV defense, with the predominant contribution of AGO2. Interestingly, the anti-PepMV activities of AGO1A, AGO5, and AGO10 only become apparent when *AGO2* is mutated. Taken together, our results prove that hierarchical actions of several AGOs are needed for the plant to build effective anti-PepMV resistance. The genetic resources created here will be valuable assets for analyzing RNAi responses triggered by other agriculturally important pathogenic viruses.

## Teaser

Hierarchical contribution of multiple Argonaute proteins is necessary for the plant to build effective anti-PepMV resistance.

## Introduction

Plants have evolved several defensive measures to combat viral infections. Of these, antiviral RNAi has been the most thoroughly characterized (Pumplin and Voinnet, 2013; Guo *et al*., 2019; Baulcombe, 2022; Lopez-Gomollon and Baulcombe, 2022; Ding, 2023) Four plant protein families have been demonstrated to play crucial roles in antiviral RNAi. RNaseIII-like enzymes (Dicer-like, DCL) recognize and subsequently convert various dsRNAs of viral origin (e.g. genomic intramolecular fold-back structures, replicative intermediates) into 2–24 nt primary viral siRNAs (vsiRNAs). This process is assisted by different double-stranded RNA-binding proteins (DRBs). Antiviral RNAi is amplified by host-encoded RNA-dependent RNA polymerases (RDRs), which use aberrant viral ssRNAs as templates for the production of dsRNAs. The resulting dsRNAs are processed by DCL–DRB complexes, yielding secondary vsiRNAs. Eventually, one strand of the vsiRNA duplexes—primary and secondary alike—is incorporated into Argonaute (AGO) protein containing RNA-induced silencing complexes (RISCs). Antiviral RISCs are able to limit the replication of the invading virus in a sequence-specific manner through a variety of mechanisms. As a highly effective countermeasure, however, viruses have acquired the ability to encode proteins that, in addition to their canonical functions, are also capable of interfering with antiviral RNAi at various steps (viral suppressors of RNA silencing, VSRs).

Studies relying on the extensive genetic resources of *Arabidopsis thaliana* have contributed greatly to elucidating the details of the molecular arms race between plants and viruses described above (Pumplin and Voinnet, 2013; Bologna and Voinnet, 2014). Yet, the use of *A. thaliana* as a virological model plant has its limitations. Most significantly, this plant species is a non-host for numerous important pathogenic viruses, which are responsible for vast amounts of damage to economically important crops. In contrast, the native Australian tobacco, *Nicotiana benthamiana*, exhibits unparalleled susceptibility to viruses (Goodin *et al*., 2008). Furthermore, since it belongs to the *Solanaceae* family, it is considered a suitable system for modeling infectious diseases of highly valuable crops such as tomatoes, potatoes, pepper, and tobacco. Nonetheless, the acceptance of *N. benthamiana* as a true model species has so far been hampered by the amphidiploid nature of its genome. Recent technical advances in the fields of next-generation sequencing and genome editing, however, may help overcome this limitation (Bally *et al*., 2018).

Pepino mosaic virus (PepMV) is a highly contagious potexvirus that poses a significant threat to tomato production worldwide (Hanssen and Thomma, 2010). The symptoms caused by PepMV are highly diverse. They range from mild fruit discoloration to ‘open fruit’, and sometimes necrosis of the leaves and stem is observed. Given the huge volume of tomato production, understanding the causes of the variable symptomatology of PepMV infection is of great interest. Sequence differences between various PepMV isolates, as well as environmental factors (temperature, light conditions, etc.) have already been demonstrated to significantly affect disease progression. Additionally, host factors, especially those directly involved in antiviral defense, may also strongly influence the severity of symptoms. This topic, however, is still unexplored due at least partly to the inability of PepMV to infect *A. thaliana*. *Nicotiana benthamiana* is a widely employed experimental host of PepMV. Recently, we have created a number of *ago* mutants of *N. benthamiana* using genome editing (Ludman *et al*., 2017; Ludman and Fátyol, 2021). Using these and several additional single and double mutants created for the study, genetic analyses were performed to assess the role of *AGO* genes in PepMV infection. We uncovered a hierarchical contribution of several AGOs to protection against PepMV, in which AGO2 played a dominant role. Interestingly, the anti-PepMV role of AGO1A, AGO5, and AGO10 is overshadowed by AGO2, and only becomes apparent when *AGO2* is mutated. Additionally, we find that the antiviral activity of the auxiliary AGOs probably relies predominantly on secondary vsiRNAs. The study presented here is the first example of the use of single and double mutants of *N. benthamiana* to conduct systematic genetic analysis, which aims to identify components of antiviral RNAi involved in protection against an emerging viral pathogen of significant economic importance.

## Materials and methods

### Plasmid construction

Plasmids were constructed using standard techniques (Sambrook *et al*., 1989). The infectious binary plasmid clone of the PepMV SP13 isolate (Aguilar *et al*., 2002) was constructed as follows: (i) wild-type *N. benthamiana* plants were inoculated with PepMV SP13 virions (kindly provided by Ioannis Livieratos); (ii) total RNA was prepared from the symptomatic systemic leaves of plants at 7 days post-inoculation (dpi); (iii) using the prepared RNA as template, the full-length cDNA copy of PepMV was amplified with suitable primers using a Long Range 2Step RT-PCR Kit (Qiagen); (iv) the cDNA was cloned into pCR-XL-2-TOPO plasmid vector using a TOPO XL-2 PCR Cloning Kit (Invitrogen); (v) the 6.4 kb *Not*I–*Spe*I restriction fragment carrying the full-length PepMV SP3 genome was cloned into a pGreen binary vector. For the generation of the *ago5* and *ago10* mutant *N. benthamiana* lines, SpCas9- or SaCas9-based editing systems were employed, respectively. The gene-specific target regions were selected using the CCTop-CRISPR/Cas9 [clustered regularly interspaced palindromic repeats (CRISPR)/CRISPR-associated protein 9] target online predictor tool (Stemmer *et al*., 2015). The single guide RNA (sgRNA)-encoding expression cassettes were inserted into the Cas9 targeting vectors as described before (Ludman *et al*., 2017; Ludman and Fátyol, 2019, 2021). Structures of all constructs were verified by sequencing. Sequences of oligonucleotides used for vector construction and sequencing are listed in Supplementary Table S1.

### Transient assays for the antiviral activity of AGOs

Agroinfiltration-based analysis of the anti-PepMV activity of *N. benthamiana* AGO proteins was performed as described earlier (Fátyol *et al*., 2016), but instead of the PVX-ΔTGB-producing infectious binary plasmid, a PepMV-producing pGreen binary construct was used (described above).

### Western analyses

Western analyses of proteins were carried out as described before (Fátyol *et al*., 2016).

### Generation of genome-edited plant lines

Genome-edited plant lines were generated using the traditional leaf disc transformation protocol detailed elsewhere (Ludman *et al*., 2017; Ludman and Fátyol, 2019, 2021).

### Plant material

Mutant *N. benthamiana* plant lines used in this study were described previously (*ago2*, *rdr6*, and *ago1a*, *ago1b* heterozygote) (Ludman *et al*., 2017; Ludman and Fátyol, 2019, 2021) or were created by genome editing in this study (*ago5* and *ago10*). Double *ago* mutant plant lines were generated by crossing the appropriate single mutants. Double homozygous *ago* mutants were identified in the F_2_ generations by sequence analyses of the appropriate segments of the *AGO* genes. The *ago2/rdr6* double mutants were obtained by crossing *ago2* homozygotes with *rdr6* heterozygotes (*rdr6* heterozygotes had to be used since *rdr6* homozygotes are sterile) (Ludman and Fátyol, 2019).

### Virus infections and RNA analyses

Virus infections and northern blot analyses were performed as described before (Fátyol *et al*., 2016; Ludman *et al*., 2017). All infections were repeated at least three times, and representative results are presented.

### Real-time quantitative reverse transcription–PCR (qRT–PCR)

RNA samples were prepared from leaf tissues as described previously (Ludman *et al*., 2017) and treated with Turbo DNase (ThermoFisher) according to the manufacturers’ instructions. DNase-treated RNA was subsequently used as template for the production of cDNA employing the High-Capacity cDNA Reverse Transcription Kit (ThermoFisher). Using the generated cDNAs as templates, qRT–PCR analyses were performed with the FastStart Essential DNA Green Master kit (Roche) according to the manufacturer’s instruction. Measurements were performed on a LightCycler 96 Instrument (Roche). The mRNA levels of *AGO* genes were determined using appropriate gene-specific primers. The measured *AGO* mRNA levels were normalized between samples by *actin* mRNA levels as internal control. PepMV RNA levels were measured with appropriate virus-specific primers and normalized as above. Sequences of oligonucleotides used for the qRT–PCR analyses are listed in Supplementary Table S1. Measurements were carried out in three biological replicates. Statistical significance of changes in mRNA levels were determined using unpaired Student’s *t*-test.

## Results

### Several AGO proteins possess the ability to target PepMV RNA

**Fig. 1.**
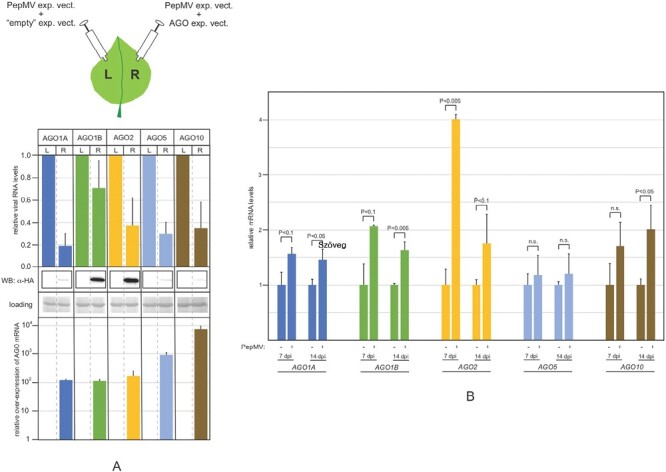
Assessment of the anti-PepMV activity of *N. benthamiana* AGO proteins. (A) For evaluation of the participation of AGO proteins in anti-PepMV defense, transient agroinfiltration-based assays were performed. An infectious clone of PepMV was introduced into *N. benthamiana* leaves either alone (one half of the leaf) or in conjunction with an expression construct producing the AGO protein of interest (the other half of the same leaf). PepMV RNA accumulation and relative overexpression of the *AGO* genes were measured in the two leaf halves by qRT–PCR. Production of the AGO proteins was also confirmed by western blotting using HA antibody. Rubisco bands of the Ponceau-stained filter are displayed as loading controls. (B) Analyses of the expression of *AGO* genes in uninfected and PepMV-infected *N. benthamiana*. *AGO* mRNA levels were measured in RNA samples prepared from the plants at 7 and 14 dpi by qRT–PCR. *AGO* mRNA levels were normalized by *actin* mRNA levels as internal controls. Measurements were performed with three biological replicates. The statistical significance of changes in *AGO* mRNA levels was determined using unpaired Student’s *t*-test. Data are given as the mean ±SD.

For the initial evaluation of the participation of AGO proteins in anti-PepMV defense, transient agroinfiltration-based assays were performed (Fig. 1A). In these functional tests, an infectious clone of a virus is introduced into *N. benthamiana* leaves either alone (one half of a leaf) or together with an expression construct that produces the AGO protein in question (the other half of the same leaf). A subsequent comparison of the levels of viral RNA in the two leaf halves makes it possible to evaluate the antiviral effect of the AGO. To carry out the assays outlined above, a cDNA copy of the full-length genomic RNA of the SP13 isolate of PepMV was cloned into a pGreen binary expression plasmid. The ability of this construct to efficiently initiate systemic viral infection after infiltration has been confirmed. The *N. benthamiana AGO* expression constructs used here had been generated and described before (Fátyol *et al*., 2016). PepMV RNA accumulation and relative overexpression of the *AGO* genes were measured by qRT–PCR. Production of the AGO proteins was also verified by western blotting. In these and all subsequent experiments, we focused our attention on those *AGO* genes that have been most widely implicated in antiviral defense, namely *AGO1* (there are two functional homeologs of *AGO1* in *N. benthamiana*, i.e. *AGO1A* and *AGO1B*) (Ludman and Fátyol, 2021), *AGO2*, *AGO5*, and *AGO10* (Silva-Martins *et al*., 2020). We found that all tested AGOs were able to reduce PepMV RNA levels, albeit to varying degrees. Interestingly, the AGOs were able to impede the accumulation of intact PepMV despite a previous report showing that in similar assays, only replication of a VSR- and movement-deficient form of potato virus X (PVXΔTGB) could be suppressed (PVX is a potexvirus related to PepMV) (Brosseau and Moffett, 2015). Regardless, the above results indicate that all tested AGO proteins possess the intrinsic ability to target and consequently reduce the accumulation of replication- and movement-competent PepMV.

### PepMV infection alters the expression of several *AGO* genes

In studies based on agroinfiltration, similar to the above, the antiviral activity of AGOs is tested under conditions where both the protein and the viral RNA are overexpressed. Consequently, it is crucial that observations made in such systems are also confirmed under biologically relevant circumstances. For a particular AGO to function in antiviral defense, it must be in the right place at the right time. Hence, we decided to monitor the expression of *AGO* genes in PepMV-infected plants. Wild-type *N. benthamiana* plants were infected with the SP-13 isolate of PepMV (Aguilar *et al*., 2002) and subsequently *AGO* mRNA levels were measured in symptomatic systemic leaves by qRT–PCR at 7 and 14 dpi (Fig. 1B). Moderate, but statistically significant induction of the two *AGO1* homeologs of *N. benthamiana* (*AGO1A* and *AGO1B*) were observed after PepMV infection at both time points compared with uninfected controls. Likewise, PepMV infection resulted in a slight, but reproducible increase of *AGO10* expression. At 7 dpi, *AGO2* was induced robustly in PepMV-infected plants compared with the uninfected cohort. At the later stage of the infection, however, *AGO2* expression declined. Although another potexvirus, PVX, strongly induced *AGO5* in both *A. thaliana* (Brosseau and Moffett, 2015) and *N. benthamiana*, no change in the gene’s expression level was observed in PepMV-infected plants at either time point.

### AGO2 is necessary for efficient protection against PepMV infection

**Fig. 2.**
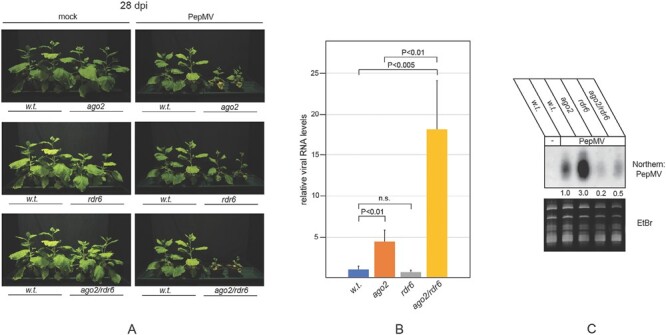
Role of *AGO2* in anti-PepMV defense. (A) *N. benthamiana* plants of the indicated genotypes were inoculated with either ‘empty’ inoculation buffer (mock) or total RNA extracted from PepMV-infected plants. Pictures of the plants were taken at 28 dpi. (B) Total RNA samples were extracted from the symptomatic systemic leaves of infected plants at 7 dpi, and PepMV gRNA levels were monitored by qRT–PCR. Viral gRNA levels were normalized by *actin* mRNA levels as internal controls. Measurements were performed with three biological replicates. The statistical significance of differences in PepMV gRNA levels was determined using unpaired Student’s *t*-test. Data are given as the mean ±SD. (C) PepMV vsiRNA levels were monitored by small RNA northern blotting. The same RNA samples were used for northern blotting as for qRT–PCRs above. The filter was hybridized with a radioactively labeled PepMV-specific probe. The relative densitometric values of the signals are displayed under the filters. As loading controls, ethidium bromide-stained gel pictures are shown. Infections were repeated at least three times, and representative results are presented.

Prompted by the above results, we proceeded to examine the antiviral role of *AGO2* in PepMV-infected *N. benthamiana*. To this end, an *ago2* mutant *N. benthamiana* plant line was used, which we had generated by genome editing and described earlier (Ludman *et al*., 2017). Wild-type and *ago2* plants were inoculated with the SP-13 isolate, and the ensuing disease was monitored for a period of 4 weeks. In wild-type plants, the virus elicited only mild symptoms, which included slight growth retardation and leaf mosaicism. In contrast, the infection resulted in severe stunting of *ago2* plants (Fig 2A). The amount of viral genomic RNA (gRNA) in symptomatic systemic leaves of infected plants was measured by qRT–PCR. In parallel, vsiRNA levels were also monitored in the same samples by northern blotting. PepMV gRNA accumulated reproducibly 4–5 times more in *ago2* plants than in the wild type (Fig. 2B), and vsiRNAs followed the same trend (Fig. 2C). Usually, AGOs use both primary and secondary vsiRNAs to mount a fully fledged antiviral response (Baulcombe, 2022; Lopez-Gomollon and Baulcombe, 2022; Ding, 2023). Since the *RDR1* gene of the LAB strain of *N. benthamiana* used in our study carries an inactivating mutation in its first exon (Yang *et al*., 2004), RDR6 is likely to be the main RDR responsible for producing secondary vsiRNAs in these plants. To assess the contribution of primary and secondary vsiRNAs to anti-PepMV defense, *rdr6* mutant plants (Ludman and Fátyol, 2019) were infected with the virus. The symptoms exhibited by the infected mutants were only slightly more severe than those of the wild-type cohort (note that the uninfected *rdr6* mutants are smaller than the age-matched wild-type plants) (Fig. 2A). To unravel potential genetic interaction between the *AGO2* and *RDR6* genes, double mutant plants were also generated by crossing the single mutants. Infection of *ago2*/*rdr6* plants with PepMV produced symptoms that were more severe than those shown by any of the single mutants (Fig. 2A). The double mutants were significantly more stunted and wilted than *ago2* plants and often necrotized by the end of the observation period. The levels of viral gRNA reflected the symptoms shown by the plants (Fig. 2B). However, the amounts of vsiRNA followed a different trend (Fig. 2C). Although the amount of gRNA in *rdr6* plants was comparable with that observed in wild-type plants, the vsiRNA level plummeted sharply (by ~80%), indicating that most of the vsiRNAs present in PepMV-infected plants are *RDR6*-dependent secondary vsiRNAs. This conclusion was also confirmed in *ago2/rdr6* double mutants, as these plants accumulated similarly significantly lower levels of vsiRNAs than wild-type plants (~50% lower), despite stockpiling much higher levels of viral gRNA (15–20 times higher than wild-type level). The synergism between *ago2* and *rdr6* was even more pronounced when the infected plants were grown at elevated temperature (24–25 °C instead of 20–21 °C) (Supplementary Fig. S1). Under these conditions, single mutants exhibited mild symptoms (as a consequence of more efficient antiviral RNAi) (Szittya *et al*., 2003), while double mutants tended to necrotize (probably due to more robust virus replication). In summary, these findings indicate that *AGO2* is a crucial component of anti-PepMV defense. Furthermore, the synergistic effects of *ago2* and *rdr6* mutations indicate that both genes contribute to anti-PepMV defense, but act at least partially independently of each other.

### Single *ago1a*, *ago5*, and *ago10* mutants do not exhibit increased susceptibility to PepMV infection

In the context of the canonical model of antiviral RNAi, the independent functions of *AGO2* and *RDR6* are relatively straightforward to interpret. On the one hand, the *RDR6*-independent antiviral activity of AGO2 is likely to be the consequence of its use of primary vsiRNAs. On the other hand, secondary vsiRNAs can interact with multiple AGOs, explaining RDR6’s contribution to anti-PepMV defense, independent of *AGO2*. To identify these *AGO* genes, PepMV susceptibility of plant lines carrying mutations in different *AGO* genes was tested. These mutants were generated previously (*ago1a* and *ago1b*) (Ludman and Fátyol, 2021) or were created for this study (*ago5* and *ago10*).

**Fig. 3.**
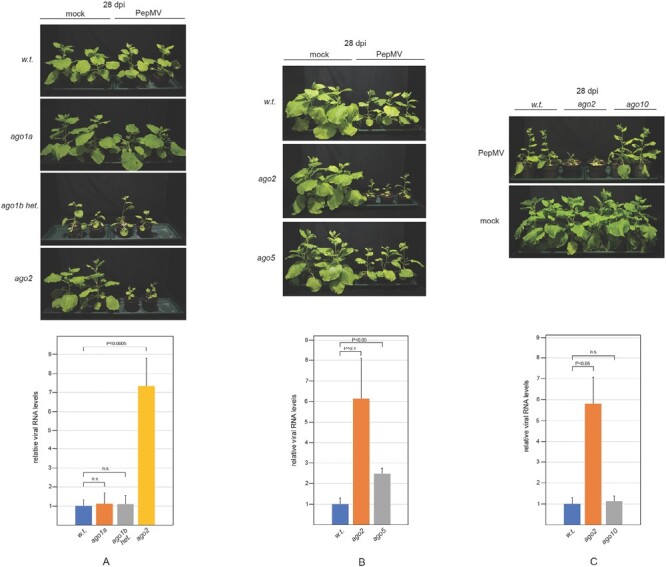
Susceptibility of individual *ago* mutants to PepMV infection. *N. benthamiana* lines carrying mutations in (A) either of the *AGO1* homeologs, (B) *AGO5*, or (C) *AGO10* were infected with PepMV. As controls, wild-type and *ago2* plants were also infected with the virus. Plants were photographed at 28 dpi, and the viral gRNA levels were monitored by qRT–PCR as described in the legend of Fig. 2. Experiments were repeated at least three times, and representative results are presented.

In addition to its role in regulating host gene expression, *AGO1* has also been consistently implicated in the antiviral defense of several plant species (Silva-Martins *et al*., 2020). In PepMV-infected *N. benthamiana*, the expression of both *AGO1* homeologs was modestly but reproducibly induced. Phenotypically, uninfected *ago1a* homozygous and wild-type *N. benthamiana* plants were indistinguishable, and both exhibited the mild symptoms described above during PepMV infection (Fig. 3A). Since *ago1b* homozygotes are unviable, *ago1b* heterozygotes had to be used to assess the contribution of this homeolog to anti-PepMV defense. These plants exhibit numerous developmental abnormalities, including stunted growth, leaf distortions, and reduced fertility. Although previously we demonstrated that *ago1b* heterozygotes were hypersusceptible to turnip crinkle virus (TCV) (Ludman and Fátyol, 2021), their challenge with PepMV did not cause obvious symptoms that were clearly attributable to the infection. Consistently, viral gRNA accumulated to comparable levels in *AGO1*-deficient and wild-type plants (Fig. 3A). In summary, under the conditions employed in our experiments, none of the mutations in *AGO1* homeologs increased the susceptibility of plants to PepMV infection.

The involvement of *AGO5* in antiviral responses has recently been reported (Brosseau and Moffett, 2015). Interestingly, we found that unlike other potexviruses, PepMV infection does not induce *AGO5* expression. Nonetheless, we decided to investigate the gene’s role in anti-PepMV defense and, to this end, *ago5* mutant *N. benthamiana* was generated by genome editing (Supplementary Fig. S2). The *ago5* plants exhibited no phenotypic alteration compared with their age-matched wild-type counterparts. Infection of *ago5* mutants with PepMV produced mild symptoms similar to those of the wild-type controls, and they accumulated viral gRNA to only an ~2-fold higher level than those controls (Fig. 3B). Combined, these findings indicate that the *AGO5* mutation does not substantially affect the plants’ susceptibility to PepMV infection.

Studies on the antiviral function of *AGO10* have so far yielded discrepant results. *AGO10* exhibits antiviral activity in inflorescence tissues of *A. thaliana* (Garcia-Ruiz *et al*., 2015), while a recent report highlights its proviral role in *N. benthamiana* (Huang *et al*., 2019). However, it should be noted that in the latter study, virus-induced gene silencing (VIGS) was used to down-regulate *AGO10* expression, which is known to have inherent limitations, especially in the context of antiviral RNAi (Ludman and Fátyol, 2019). To overcome this confounding issue, we created *ago10 N. benthamiana* using genome editing (Supplementary Fig. S3). Phenotypically, the uninfected *ago10* mutants were indistinguishable from wild-type plants. PepMV infection resulted in mild symptoms in *ago10* plants comparable with those of the wild-type cohort (Fig. 3C). Accordingly, viral gRNA accumulated to the same level in both groups. In summary, none of the individual mutations of *AGO1* homeologs or of *AGO5* or *AGO10* resulted in increased susceptibility of *N. benthamiana* to PepMV infection.

### *AGO2* masks the antiviral effects of other *AGO* genes

**Fig. 4.**
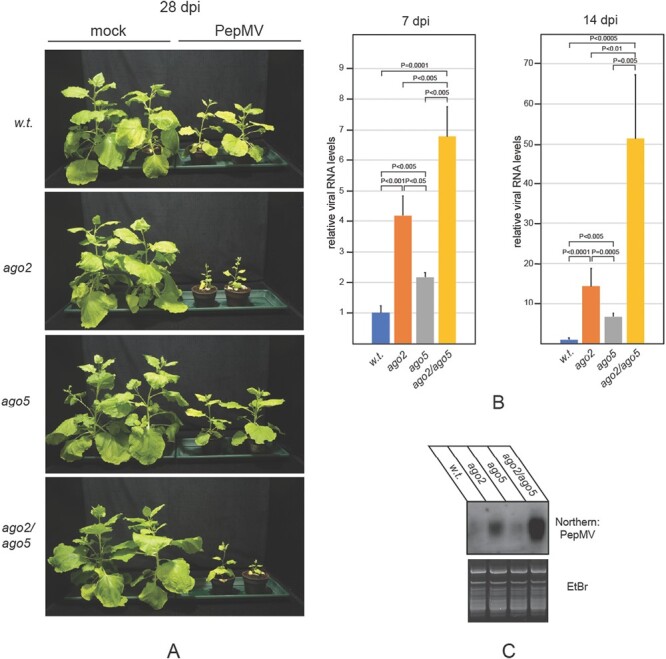
Susceptibility of *ago2/ago5* double mutant *N. benthamiana* to PepMV infection. (A) *N. benthamiana* plants of the indicated genotypes were inoculated with either ‘empty’ inoculation buffer (mock) or total RNA extracted from PepMV-infected plants. Pictures of the plants were taken at 28 dpi. Total RNA samples were extracted from the symptomatic systemic leaves of infected plants at the indicated time points. (B) Viral gRNA levels and (C) vsiRNA levels were monitored as described in the legend of Fig. 2. Experiments were repeated at least three times, and representative results are presented.

The resistance of single *ago* mutants to PepMV infection, described above, was somewhat unexpected. One plausible explanation is that the strong antiviral activity of the *AGO2* gene may be able to mask the contribution of other *AGO* genes to antiviral protection. *AGO2*, among other mechanisms, may be able to achieve this by inhibiting the expression of other *AGO* genes. To test this hypothesis, we compared the expression levels of *AGO* genes in PepMV-infected *ago2* mutants with those in wild-type plants (Supplementary Fig. S4). Mutation of *AGO2* led to no change in the expression of any of the *AGO1* homeologs or *AGO10*, regardless of PepMV infection. However, challenging *ago2* and *ago2/rdr6* plants with PepMV, unlike wild-type plants, resulted in a robust induction of *AGO5*, suggesting that *AGO5* may indeed be involved in anti-PepMV defense. To investigate this possibility further, *ago2*/*ago5* double mutants were generated by crossing the single mutants, and subsequently these plants were infected with the virus. In general, *ago2*/*ago5* plants produced symptoms similar to those of *ago2* plants (Fig. 4A). However, the double mutants accumulated significantly more viral gRNA than any of the single mutants (Fig. 4B). This was already observable at 7 dpi, but it become even more evident at 14 dpi, by which time *ago2*/*ago5* plants contained ~3-fold more PepMV gRNA than *ago2* mutants (~50 times higher than wild-type plants). The levels of vsiRNAs followed those of the viral gRNA (Fig. 4C).

**Fig. 5.**
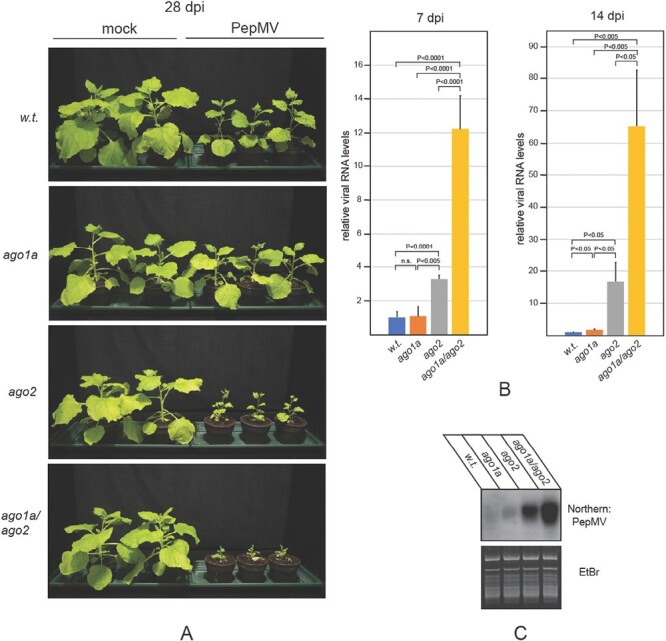
Susceptibility of *ago1a/ago2* double mutant *N. benthamiana* to PepMV infection. (A) *N. benthamiana* plants of the indicated genotypes were inoculated with either ‘empty’ inoculation buffer (mock) or total RNA extracted from PepMV-infected plants. Pictures of the plants were taken at 28 dpi. Total RNA samples were extracted from the symptomatic systemic leaves of infected plants at the indicated time points. (B) Viral gRNA levels and (C) vsiRNA levels were monitored as described in the legend of Fig. 2. Experiments were repeated at least three times, and representative results are presented.

Although the expression of the *AGO1* homeologs was not affected by the *AGO2* mutation, we also sought to test their participation in anti-PepMV defense. The fertility of *ago1b* heterozygous plants is strongly reduced. As a likely consequence, we were not able to introgress the mutant *ago1b* allele into *ago2* homozygotes. Hence, we could not test the role of *AGO1B* in antiviral defense, in the absence of *AGO2*. In contrast, by crossing the appropriate single mutants, the creation of *ago1a*/*ago2* double homozygous plants was problem free. Infection of these plants with PepMV resulted in more severe symptoms than those exhibited by *ago2* mutants (Fig. 5A). At 7 dpi, viral gRNA had already accumulated an ~3-fold higher level in *ago1a*/*ago2* plants than in *ago2* mutants, and elevated virus levels persisted to at least 14 dpi (Fig. 5B). The levels of vsiRNAs followed the same trend as those of viral gRNA (Fig. 5C).

**Fig. 6.**
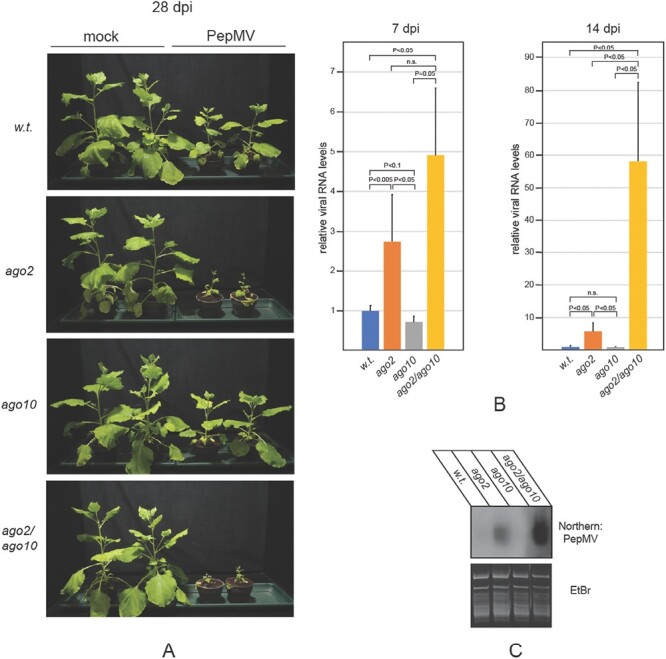
Susceptibility of *ago2/ago10* double mutant *N. benthamiana* to PepMV infection. (A) *N. benthamiana* plants of the indicated genotypes were inoculated with either ‘empty’ inoculation buffer (mock) or total RNA extracted from PepMV-infected plants. Pictures of the plants were taken at 28 dpi. Total RNA samples were extracted from the symptomatic systemic leaves of infected plants at the indicated time points. (B) Viral gRNA levels and (C) vsiRNA levels were monitored as described in the legend of Fig. 2. Experiments were repeated at least three times, and representative results are presented.

Finally, to examine the contribution of *AGO10* to anti-PepMV defense in the absence of *AGO2*, *ago2/ago10* double mutants were also generated by crossing *ago2* and *ago10* plants. The double mutants tended to show more severe symptoms than *ago2* plants and consistently accumulated more viral gRNA (Fig. 6A, B). As above, the accumulation of vsiRNAs showed the same pattern as that of viral gRNA (Fig. 6C). In summary, *AGO1A*, *AGO5*, and *AGO10* are all involved in anti-PepMV defense, but their effects only become apparent when *AGO2* is dysfunctional.

## Discussion

AGOs are essential components of plant antiviral defense. AGO1, the founding member of the plant AGO protein family, beside its numerous functions in development, hormonal regulation, biotic, and abiotic stress responses, was also the first one to be implicated in antiviral RNAi. The activity of AGO1 is often neutralized by virus-encoded VSRs, which provides solid evidence for the molecule’s involvement in antiviral protection (Silva-Martins *et al*., 2020; Ding, 2023). Besides AGO1, the antiviral activity of AGO2 has also been widely documented (Harvey *et al*., 2011; Jaubert *et al*., 2011; Scholthof *et al*., 2011; Wang *et al*., 2011; Alvarado and Scholthof, 2012; Carbonell *et al*., 2012; Zhang *et al*., 2012; Odokonyero *et al*., 2015; Fátyol *et al*., 2016; Alazem *et al*., 2017; Ludman *et al*., 2017; Paudel *et al*., 2018). Initially, it was reported that AGO2 complements the antiviral activity of AGO1, by forming a second layer of protection against viruses that are able to suppress AGO1 function. At the molecular level, this is achieved by VSR-mediated inhibition of the AGO1/miR403 regulatory circuit, resulting in increased expression of *AGO2* (Harvey *et al*., 2011). However, it was later discovered that AGO2 could also act against viruses that were not known to target AGO1 (Ma *et al*., 2015; Ludman and Fátyol, 2021). Analysis of the RNAi response elicited by PepMV infection is not only consistent with these reports, but also sheds new lights on them, demonstrating that the strong antiviral activity of AGO2 may even mask the antiviral effects of other AGOs.

**Fig. 7.**
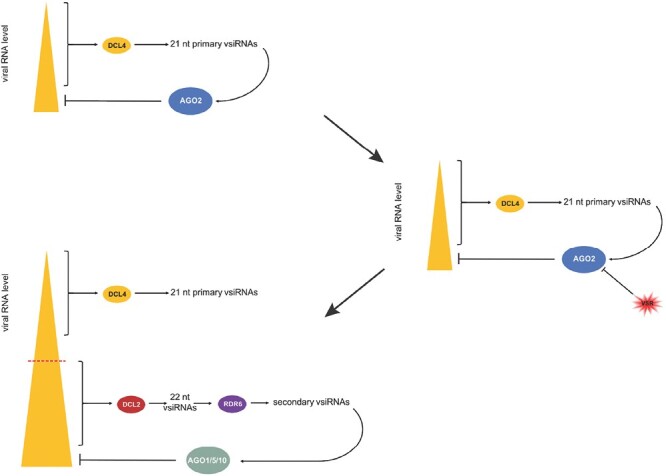
Model of the sequential hierarchical contribution of AGO proteins to antiviral RNAi. See details in the text.

In transient assays, all AGOs generally implicated in antiviral RNAi were capable of reducing the accumulation of PepMV RNA (Fig. 1A). Remarkably, however, only the *ago2* mutant plants exhibited hypersensitivity to PepMV infection, while individual mutations in the *AGO1A*, *AGO1B*, *AGO5*, or *AGO10* genes did not significantly affect disease progression (Fig. 3). The robust induction of *AGO2* expression in PepMV-infected plants also underscores its importance for protection against PepMV (Fig. 1B). Efficient antiviral RNAi generally relies on both primary and secondary vsiRNAs (Pumplin and Voinnet, 2013; Guo *et al*., 2019; Baulcombe, 2022; Lopez-Gomollon and Baulcombe, 2022; Ding, 2023). In the LAB strain of *N. benthamiana* used here, the production of secondary vsiRNAs is presumed to be *RDR6* dependent (Yang *et al*., 2004). Consequently, it was unexpected that symptoms in PepMV-infected *rdr6* mutants were quite mild, and not significantly more severe than those of wild-type plants (Fig. 2A). Our findings were particularly surprising because, despite similar accumulation of viral gRNA in the two groups of plants, the vsiRNA level dropped sharply in *rdr6* mutants compared with wild-type controls (Fig. 2B, C). This observation indicates that in PepMV-infected plants the majority of vsiRNAs are produced in an *RDR6*-dependent fashion. Therefore, the relative resistance of *rdr6* plants to PepMV infection, as opposed to the hypersensitivity of *ago2* plants to the virus, clearly shows that the strong anti-PepMV effect of AGO2 is predominantly dependent on primary vsiRNAs. Nonetheless, *ago2* and *rdr6* mutations exhibit strong synergism with each other, as indicated by the potent exacerbation of symptoms exhibited by PepMV-infected *ago2/rdr6* double mutants compared with single mutants (Fig. 2A). This result implies that the two genes contribute to anti-PepMV defense largely independently of each other. In the context of the prevailing model of antiviral RNAi, this finding is most consistent with the assumption that *RDR6*-dependent secondary vsiRNAs predominantly interact with AGOs other than AGO2, complementing the plant’s anti-PepMV defense. Hence, it was particularly interesting to find that of the single *ago* mutants tested, only *ago2* plants displayed heightened sensitivity to PepMV infection. A plausible explanation for this apparent discrepancy is that the strong antiviral activity of *AGO2* may be able to mask the similar activities of other *AGO* genes. Consistently, in *ago2* mutants—unlike wild-type plants—PepMV infection resulted in strong induction of the *AGO5* gene (Supplementary Fig. S4), and *ago2*/*ago5* double mutants also had an increased accumulation of viral gRNA compared with the single mutants (Fig. 4B). Although the *ago2* mutation did not affect the expression levels of any of the *AGO1* homeologs or *AGO10* (Supplementary Fig. S4), both *ago1a*/*ago2* and *ago2*/*ago10* double mutants exhibited increased symptom severity and virus accumulation during PepMV infection relative to the single mutants (Figs 5, 6). Taken together, these results strongly support the idea that in addition to *AGO2*, other *AGO* genes are also needed for the plant to build effective anti-PepMV resistance. The need for multiple *AGO* genes for an effective antiviral response can be best understood by assuming that the *AGO* genes act hierarchically in a sequential fashion, depending on the cellular concentration of the viral RNA (Fig. 7). This model is also compatible with the proposed competition between DCL2 and DCL4 for virus-derived dsRNAs (precursors of vsiRNAs) during viral infection (Bouché *et al*., 2006). Briefly, in the early stages of infection, when the viral RNA level is still low, predominantly DCL4 would produce limited amounts of 21 nt vsiRNAs, which would largely be used by the primary AGO—in this case AGO2—to limit the replication of the virus. If this step proves insufficient to suppress infection (e.g. due to the action of a VSR) and, as a result the viral RNA level continues to rise, then, in addition to DCL4, DCL2 would also engage virus-derived dsRNAs, producing 22 nt vsiRNAs. These vsiRNAs, in turn, would trigger *RDR6*-dependent secondary vsiRNA production and rapid amplification of the antiviral RNAi (Sakurai *et al*., 2021; Yoshikawa *et al*., 2021). At this stage of the infection, the contribution of the auxiliary *AGO* genes such as *AGO1*, *AGO5*, and *AGO10* to the antiviral response would also become substantial. The increased contribution of these *AGO* genes to antiviral defense may be due to their elevated expression (e.g. *AGO5*) and/or preferential use of secondary vsiRNAs. The hierarchical actions of multiple AGOs in antiviral RNAi, proposed by this model, may have several advantages, as it would (i) result in better protection against viruses that produce VSRs targeting specific AGOs; (ii) allow more efficient utilization of the diverse vsiRNA pool produced during viral infection; and (iii) provide more effective and economical protection in the case of mixed viral infection.

For genetic analysis of the antiviral RNAi response elicited by PepMV infection, a series of single and double mutants of *N. benthamiana* were employed. These mutants were created using CRISPR/Cas9-mediated genome editing. Like PepMV, many important pathogenic viruses are unable to infect the model plant *A. thaliana*, complicating the identification of host factors that influence disease progression. Advances in next-generation sequencing and genome editing in recent years, however, could help solve this problem by elevating *N. benthamiana* to the level of a bona fide model species, allowing studies such as the one presented here to be conducted.

## Supplementary data

The following supplementary data are available at *JXB* online.

Fig. S1. Analyses of the synergism between *ago2* and *rdr6* mutations during PepMV infection.

Fig. S2. Inactivation of *N. benthamiana AGO5* by CRISPR/SaCas9.

Fig. S3. Inactivation of *N. benthamiana AGO10* by CRISPR/SpCas9.

Fig. S4. Analyses of the expression of *AGO* genes in PepMV-infected mutant and wild-type *N. benthamiana* plants.

Table S1. Sequences of oligonucleotides used in this study.

erad327_suppl_Supplementary_Material

## Data Availability

All data supporting the findings of this study are available within the paper and within its supplementary data published online.

## Abbreviations

AGO: argonaute
CRISPR: clustered regularly interspaced short palindromic repeats
DCL: Dicer-like
dpi: days post-inoculation
DRB: double-stranded RNA-binding protein
gRNA: genomic RNA
PepMV: pepino mosaic virus
PVX: potato virus X
RDR: RNA-dependent RNA polymerase
vsiRNA: viral siRNA
VSR: viral suppressor of RNA silencing
