# Supplementary material for: Hierarchical contribution of Argonaute proteins to antiviral protection
Source: J Exp Bot. 2023 Aug 21;74(21):6760–72. doi: 10.1093/jxb/erad327 (PMC10662219; doi:10.1093/jxb/erad327)
Supplement: erad327_suppl_Supplementary_Material [file erad327_suppl_supplementary_material.pdf]

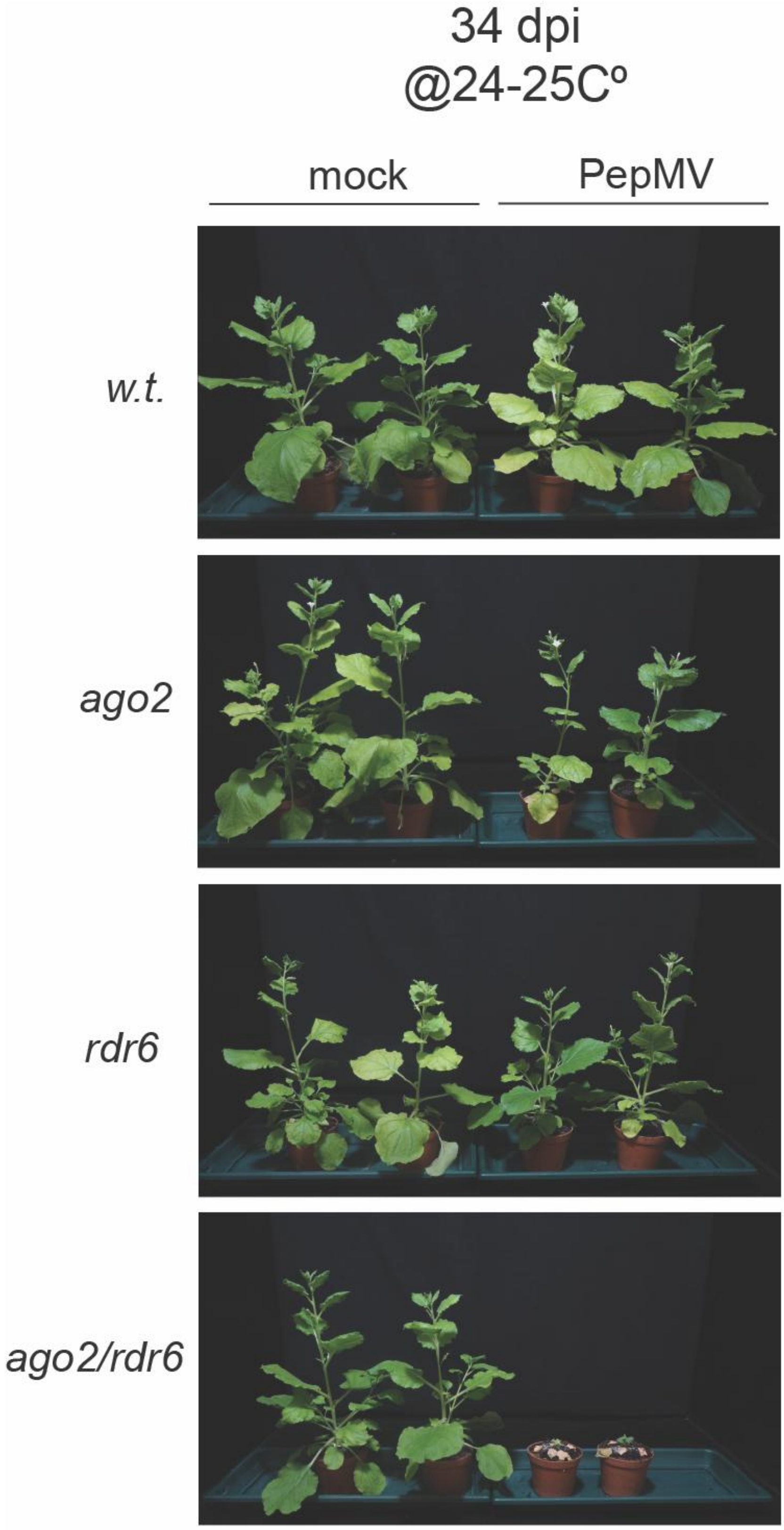

Supplementary Figure S1. Analyses of the synergism between ago2 and rdr6 mutations during PepMV infection. N. benthamiana plants of the indicated genotypes were inoculated either with "empty" inoculation buffer (mock) or total RNA extracted from PepMV-infected plants. The infected plants were grown at 24-25oC instead of 20-21oC. Pictures of the plants were taken at 34 dpi.

Supplementary Figure 1

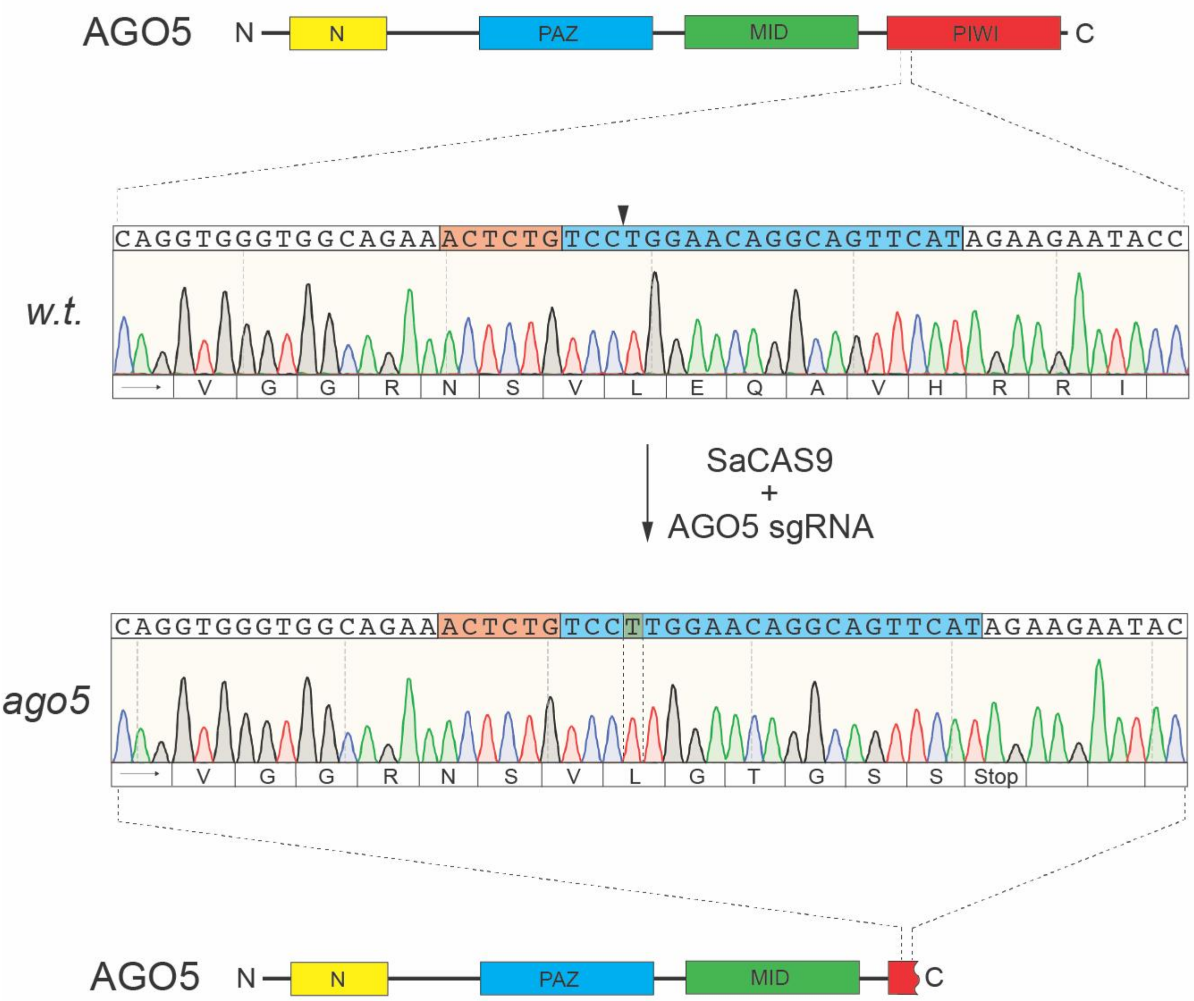

Supplementary Figure S2. Inactivation of N. benthamiana AGO5 by CRISPR/SaCas9. Sequencing chromatogram of a PCR fragment amplified from a plant carrying wild-type AGO5 alleles is shown on the top. Target region and protospacer adjacent motif are highlighted by blue and pink, respectively. SaCas9 cleavage site is indicated by arrowhead. Sequencing chromatogram of a PCR fragment amplified from an ago5 mutant plant is shown at the bottom. ago5 plants are homozygous, carrying a single T insertion in both alleles. Inserted T is highlighted by green. Sketches of wild-type (top) and mutant AGO5 proteins (bottom) are also shown.

Supplementary Figure 2

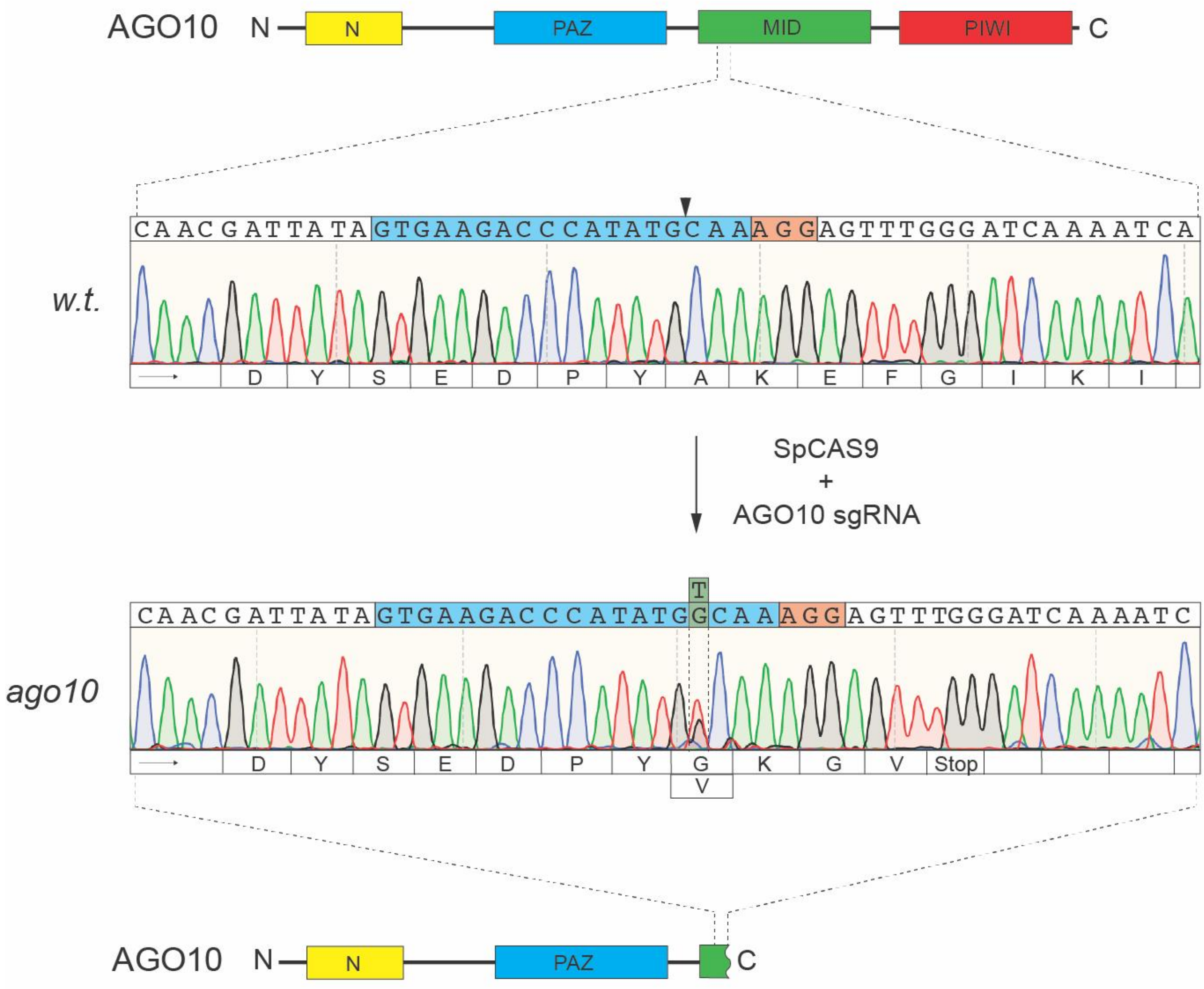

Supplementary Figure S3. Inactivation of N. benthamiana AGO10 by CRISPR/SpCas9. Sequencing chromatogram of a PCR fragment amplified from a plant carrying wild-type AGO10 alleles is presented on the top. Target region and protospacer adjacent motif are highlighted by blue and pink, respectively. SpCas9 cleavage site is indicated by arrowhead. Sequencing chromatogram of a PCR fragment amplified from an ago10 mutant plant is shown at the bottom. ago10 plants are biallelic, one allele carrying a T while the other a G insertion. Insertions are highlighted by green. Sketches of wild-type (top) and mutant AGO10 proteins (bottom) are also shown.

Supplementary Figure 3

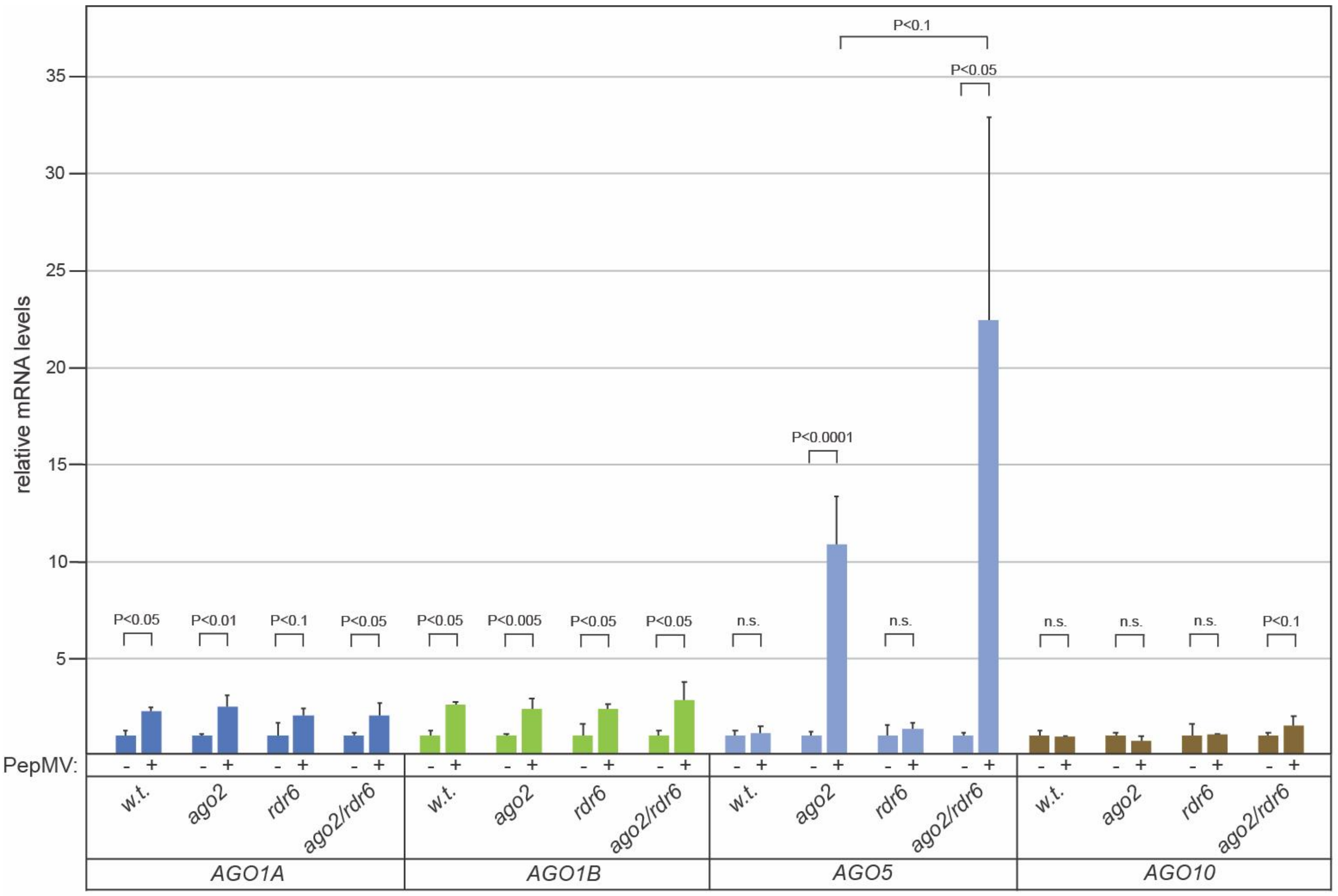

Supplementary Figure S4. Analyses of the expression of AGOs in PepMV infected mutant and wild-type N. benthamiana plants. AGO mRNA levels were measured in RNA samples prepared from the plants at 7 dpi by qRT-PCR. AGO mRNA levels were normalized by actin mRNA levels as internal control. Measurements were performed with three biological replicates. The statistical significance of changes in AGO mRNA levels was determined using unpaired Students' t test. Data given as mean ± SD.

Supplementary Figure 4

oligo name	sequence	use
NbAct(11-35)Fw	GAG AGG ATA TTC AGC CAC TTG TCT G	NbActin-specific primer for qRT-PCR
NbAct(160-138)Rev	CAT CTT TCT GAC CCA TAC CCA CC	NbActin-specific primer for qRT-PCR
NbAGO1A-3'UTR-Fw	GAA GTT CTC GCT AAA CCG	NbAGO1A 3'UTR-specific primer for qRT-PCR
NbAGO1A-3'UTR-Rev	GAC CCA ATT TGT GCT AGA C	NbAGO1A 3'UTR-specific primer for qRT-PCR
NbAGO1B-3'UTR-Fw	GCT ATA GTT TGG ACT TTA G	NbAGO1B 3'UTR-specific primer for qRT-PCR
NbAGO1B-3'UTR-Rev	GCT TTC CGG CAT CAT AG	NbAGO1B 3'UTR-specific primer for qRT-PCR
NbAGO2(1789-1809)Fw	GTT CCA CCA CGA GAG AGA AGG	NbAGO2-specific primer for qRT-PCR
NbAGO2(1939-1917)Rev	GCT TCA AAT CAG GGG TAG GAA GG	NbAGO2-specific primer for qRT-PCR
NbAGO5(302-321)Fw	CTG GTG TAC AAC CGC TAC AG	NbAGO5-specific primer for qRT-PCR
NbAGO5(447-428)Rev	GTG ATG CAG ATC CCG ATC AG	NbAGO5-specific primer for qRT-PCR
NbAGO10(118-136)Fw	CCA CCA ATC ACA GAA CCC C	NbAGO10-specific primer for qRT-PCR
NbAGO10(243-223)Rev	CCT CAT AAA GGC CTC ACC TTG	NbAGO10-specific primer for qRT-PCR
SaCas9 AGO5 T4-U	ATT GAT GAA CTG CCT GTT CCA GGA	NbAGO5 sgRNA target 2 oligo
SaCas9 AGO5 T4-L	AAA CTC CTG GAA CAG GCA GTT CAT	NbAGO5 sgRNA target 2 oligo
AGO5-SaTar-Fw	GCA CCG CAA TAT TGA AGT AAT G	amplification of NbAGO5 target region
AGO5-SaTar-Rev	CTT CGA AAC GCA ATC AGT AAC	amplification of NbAGO5 target region
PrimerF2-A10-2	GTG AAG ACC CAT ATG CAA GTT TTA GAG CTA GAA ATA GC	generation of NbAGO10-specific sgRNA expression cassette
PrimerR1-A10-2	TTG CAT ATG GGT CTT CAC CAA TCA CTA CTT CGT CTC T	generation of NbAGO10-specific sgRNA expression cassette
PrimerF1	CGC GAC GTC AGA AAT CTC AAA ATT CCG	generation of NbAGO10-specific sgRNA expression cassette
PrimerR2	CGC GAC GTC TAA TGC CAA CTT TGT ACA	generation of NbAGO10-specific sgRNA expression cassette
A10-2-Fw	GTG CAA CAC ATC TAC ATG GG	amplification of NbAGO10 target region
A10A-Rev	GAT ATT GGG CCT TTT TAT TTT GT	amplification of NbAGO10 target region
NBAGO2gF	GAG GTA GCA ATA TGG AAC TTA CG	amplification of NbAGO2 target region
NBAGO2gR	CAT GAT AGT GAG TAG GCT TGC TAG	amplification of NbAGO2 target region
NbRDR6(254-276)-Fw	CAG AGT CCG CAA AGT ATG CTC TG	amplification of NbRDR6 target region
NbRDR6(901-887)-Rev	GGT TAC ACA TTT CTA CCA TGG GAA C	amplification of NbRDR6 target region
PepMV(5725-5744)-Fw	CAA TAC AGC TCC TAG TCT CA	PepMV-specific primer for qRT-PCR
PepMV(5891-5873)-Rev	CAT ATG CAC GAG CTA GAT C	PepMV-specific primer for qRT-PCR

Supplementary Table S1. Sequences of oligonucleotides used in this study.
